# Prescribing Patterns of Psychotropic Medications for Common Psychiatric Disorders in a Mental Health Hospital in Tabuk

**DOI:** 10.7759/cureus.54927

**Published:** 2024-02-26

**Authors:** Mostafa A Ali, Palanisamy Amirthalingam, Vinoth-Prabhu Veeramani, Abdulmajeed M Alasiri, Meshal N Alsalimi, Fahad Alatawi, Mutlaq M Albalawi

**Affiliations:** 1 Pharmacy Practice Department, Faculty of Pharmacy, University of Tabuk, Tabuk, SAU; 2 Department of Clinical Pharmacy, Faculty of Pharmacy, Assiut University, Assiut, EGY; 3 Pharmacy, University of Tabuk, Tabuk, SAU; 4 Pharmacy, Eradah Mental Health Hospital, Tabuk, SAU

**Keywords:** schizophrenia, psychotropic medication, prescribing pattern, polypharmacy, mental health

## Abstract

Introduction: Mental health disorders are often chronic and disabling conditions that impact different populations, irrespective of age, cultural background, or socioeconomic status. This study aimed to describe the prescribing pattern of psychotropic medications in patients with psychiatric disorders.

Methods: This research was a retrospective investigation of psychotropic medication prescribing patterns and conducted at Eradah Mental Health Hospital at Tabuk City, Saudi Arabia. The data were extracted from the electronic medical database of adult patients presenting to the outpatient and inpatient clinics of a mental health hospital between 2020 and 2023. The diagnosis of psychiatric disorders was identified using the International Classification of Diseases 10th Revision (ICD-10) codes version 2019 for mental and behavioral disorders (F00 to F99).

Results: The electronic medication records of 526 patients were reviewed, of whom 510 (97%) were Saudi nationals, 368 (69.9%) were males, 235 (44.7%) were between 31 and 40 years of age, and 233 (44.3%) were outpatients. The most common psychiatric diagnoses were schizophrenia (33.3%), followed by bipolar affective disorder (16.9%). A total of 2,153 medication orders for psychotropic medications were quantified. Second-generation antipsychotics (SGAs) and first-generation antipsychotics (FGAs) were the top prescribed medication classes (1,290, 60%), followed by mood stabilizers (324, 15%). More than one-half of the patients (282, 53.6%) received a combination of antipsychotic and mood stabilizers, and 155 (29.5%) received a combination of antipsychotic and antidepressant medications. No significant differences were observed in prescribing for males and females.

Conclusion: Schizophrenia was highly prevalent among adult patients who sought mental healthcare. Polypharmacy of psychotropic medication was common depending on the symptoms' severity and comorbid psychiatric diseases.

## Introduction

Psychiatric disorders are disabling and generally chronic diseases with common features of hallucinations, delusions, and impairment of cognitive and social functioning [1]. Social functioning deficits are a common feature of most psychiatric disorders that require additional treatment intervention for many conditions [2].

According to the 2015 Global Burden of Disease (GBD) report, drug use disorders, depressive disorders, and anxiety disorders rank as the third, fourth, and sixth most significant contributors to disability in the Kingdom of Saudi Arabia (KSA), respectively [3]. The Saudi National Mental Health Survey (SNMHS) was a large-scale epidemiological survey carried out from 2011 to 2016 [4]. It was a cross-sectional community-based, psychiatric epidemiological household survey that targeted 4,004 Saudi citizens aged 15 to 65, selected to represent the national population [4]. The technical report of the survey revealed that 34% of Saudis had diagnostic criteria for a mental health disorder sometime in their life. Anxiety disorders (11.9%), followed by attention-deficit/hyperactivity disorder (8%), and major depressive disorder (6%) were the most prevalent mental health conditions in the KSA across the lifetime.

The survey also reported that the Saudi mental health legislation allocated 4% of total healthcare spending to mental disorders compared to an average of less than 2% worldwide. However, this outlay was lower than those in other high-income countries, where a median of 6% of total healthcare spending is assigned to mental disorders. The country boasts a mental health professional rate of 19.4 per 100,000 population, surpassing the global average of 6.6 per 100,000, yet it is significantly behind the median rate observed in high-income countries, which stands at 64.3 per 100,000 population [4].

Therapeutic interventions in psychosis have been proven to decrease the severity of the illness, lower hospitalization rates, and improve social functioning, such as participation in school or occupation. Antipsychotics, antidepressants, and anxiolytics are the mainstay psychotropic medications for the management of psychological distress, mood disorders, and anxiety [5]. Antipsychotic medications are a key component and first-choice pharmacotherapy for those experiencing schizophrenia, and some antipsychotics are approved for other psychotic disorders, such as bipolar disorders and refractory depression [5,6]. Antipsychotic medications are broadly classified into two categories, namely, first-generation antipsychotics (FGAs) and second-generation antipsychotics (SGAs), which are the mainstay treatment for psychiatric disorders, such as schizophrenia, despite the variability in efficacy and adverse effects [5]. SGAs are known to induce metabolic side effects, such as obesity, diabetes mellitus, and dyslipidemia, whereas FGAs are more likely associated with extrapyramidal symptoms (EPS) and rarely neuroleptic malignant syndrome [7].

Pharmacological treatment guidelines for antipsychotic use in psychosis suggest that the initial choice of medications should prioritize their side-effect profile with doses at the lower end of the range [6]. However, a significant proportion of individuals with psychosis fail to attain an adequate clinical response. Guidelines recommended a shift toward the use of SGAs as the initial treatment for first-episode psychosis [8-10]. Clozapine is the treatment of choice for refractory schizophrenia and for patients with a high risk of suicide attempts despite other treatments [8].

Moreover, compared to the general population, patients with psychiatric disorders are more likely to experience premature death due to poor health outcomes, including mortality from cardiometabolic side effects of antipsychotic drugs [11]. Therefore, rational and evidence-based prescribing is essential to ensure the safe, effective, and economical use of medicines and adherence to therapy.

Current studies of psychiatric morbidity and prescribing patterns carried out in the KSA focused mainly on specific populations with limited samples, such as patients in hospitals, primary healthcare centers, or student populations [4]. Limited data are available about prescribing patterns of psychotropic medications, rational prescribing with concurrent diseases, and associated adverse effects in the Tabuk region. The primary aim of the study was to describe the pattern of psychotropic medication use in patients who are treated in a mental health hospital using electronic medical records.

## Materials and methods

Study design and setting

This was a retrospective investigation of psychotropic prescribing patterns and conducted at Eradah Mental Health Hospital at Tabuk City, Saudi Arabia. The clinical and medication data were extracted from the electronic medical database and electronic medication records (EMRs) between 2020 and 2023. This article was drafted using the Strengthening the Reporting of Observational Studies in Epidemiology (STROBE) guidelines for reporting cross-sectional studies.

The study was conducted at a mental health hospital affiliated with the Ministry of Health. It is a governmental mental health specialized hospital providing treatment interventions for addiction and healthcare management for all mental health disorders, including psychiatric treatment and social care. The hospital was established more than 20 years ago and comprises buildings for emergencies, intensive care units, outpatient clinics, and the supportive services of pharmacies, laboratories, and radiology. The hospital is the only specialized hospital in the region concerned with treating psychiatric and neurological disorders. The majority of Saudi citizens are medically insured through governmental hospitals, and they receive their maintenance therapy for psychiatric and neurological disorders from the outpatient pharmacy of the mental health hospital.

Data collection procedure

Retrospective data were extracted from the EMRs of all adult patients diagnosed with psychiatric disorders and admitted to outpatient and inpatient clinics at the mental health hospital.

The sample size was estimated using the Raosoft Sample Size Calculator (http://www.raosoft.com/samplesize.html) [12] based on a margin of error of 5%, a confidence level of 95%, a population size of the province above 18 years old of 560,467 according to the General Authority for Statistics in 2022, and a response distribution of 50%. The calculated sample size was 384.

Outcomes

The diagnosis of psychiatric disorders was identified using the International Classification of Diseases 10th Revision (ICD-10) codes version 2019 (Chapter V: Mental and behavioral disorders (F00- F99), https://icd.who.int/browse10/2019/en#/V), which is the classification tool adopted by the hospital to identify the diagnosis of mental disorders.

Children less than 18 years and pregnant women were excluded from the study (F50-59, F80-89, F90-98). The documented patients' demographic and disease data, such as age, gender, diagnosis, symptoms, laboratory results, length of illness, and comorbid diseases, were collected (https://forms.gle/yQduqTnSaBGAsuN29).

Prescribing patterns of psychotropic medications: regular medication data including generic names, doses, and frequency of prescribed medications were retrieved and extracted from the medication charts of outpatients and the medication administration records of inpatients.

Psychotropic medications were classified into the following classes: (a) antipsychotics (first-generation and second-generation); (b) antidepressants (selective serotonin reuptake inhibitors (SSRIs), serotonin and norepinephrine reuptake inhibitors (SNRIs), tricyclic antidepressants, and monoamine oxidase inhibitors); (c) mood stabilizers; and (d) anxiolytics (short-acting and long-acting benzodiazepines).

The prescriptions for individual medications were grouped according to inpatient and outpatient settings. Psychotropic polypharmacy was defined as the concurrent prescribing of ≥2 psychotropic medications from the same or different pharmacological class [13-15].

Statistical analysis

The data were analyzed using the IBM SPSS Statistics for Windows, version 22 (released 2013; IBM Corp., Armonk, New York, United States). Standard descriptive statistics were used to describe the baseline demographic and disease-related characteristics of the patients and general trends in medication prescribing. The frequencies and percentages of demographic characteristics, comorbidities, psychiatric diagnoses, and prescribed medications were illustrated. The median and range (minimum and maximum) of the prescribed doses of individual psychotropic medications and median and quarterly ranges of the number of prescribed medications per patient were reported. The difference in medication use between inpatient and outpatient settings was tested using the chi-square or Fisher’s exact test (as appropriate). The difference in the number of prescribed medications between male and female patients was tested using the two-sample Wilcoxon rank-sum (Mann-Whitney) test. A p-value of less than 0.05 was considered statistically significant.

## Results

Demographic and clinical characteristics

The demographic and clinical characteristics of the study population are described in Table 1. The EMRs of 526 patients were reviewed between 2020 and 2023, of whom 510 (97%) were Saudi nationals, 368 (69.9%) were males, 235 (44.7%) were between 31 and 40 years of age, and 233 (44.3%) were outpatients (Table 1). The most common identified psychiatric diagnoses were schizophrenia (33.3%), followed by bipolar affective disorder (16.9%). The anxiety disorders (4.9 %) included panic disorder, social phobia, post-traumatic stress disorder, and obsessive-compulsive disorder. The number of patients with unspecified ICD-10 diagnoses of psychotic disorder in their medical records was 161 (30.6%). Some patients suffered from comorbid diseases, such as diabetes (5.7%) and hypertension (2.5%).

**Table 1:** Patients' baseline demographic and clinical characteristics ALT: alanine transaminase, AST: aspartate aminotransferase, BUN: blood urea nitrogen, HDL: high-density lipoprotein

Item	Frequency (%) n= 526
Gender	
Male	368 (69.9)
Female	158 (30.1)
Age	
18-30	150 (28.5)
31-40	235 (44.7)
41-60	104 (19.8)
>60	37 (7.0)
Clinic, outpatient	233 (44.3)
Duration of psychotic disease (years) 1-3	519 (98.7)
>3	7 (1.3)
Psychiatric diagnoses	
Anxiety disorder	26 (4.9)
Bipolar affective disorder	89 (16.9)
Dementia	8 (1.5)
Depression (major depressive)	46 (8.7)
Emotional unstable personality disorders	8 (1.5)
Mental retardation	40 (7.6)
Schizophrenia	175 (33.3)
Unspecified mental and behavioral disorders	161 (30.6)
Comorbidities	
Diabetes mellitus	30 (5.7)
Hypertension	13 (2.5)
Epilepsy	9 (1.7)
Alzheimer's disease	6 (1.1)
Laboratory investigations for inpatient (serum)	Mean (SD)
Creatinine (µmol/L)	78.4 (25.7)
Blood glucose (mmol/L)	6.4 (2.3)
AST (U/L)	23.6 (13.3)
ALT (U/L)	22.0 (13.9)
Bilirubin (µmol/L)	8.6 (7.1)
Protein (g/L)	68.2 (5.8)
BUN (mmole/l)	3.9 (1.6)
Uric acid (mg/dL)	6.8 (5.9)
Cholesterol (mmol/L	4.7 (5.7)
HDL	1.3 (0.4)

Prescribing pattern of psychotropic medications

**Figure 1:**
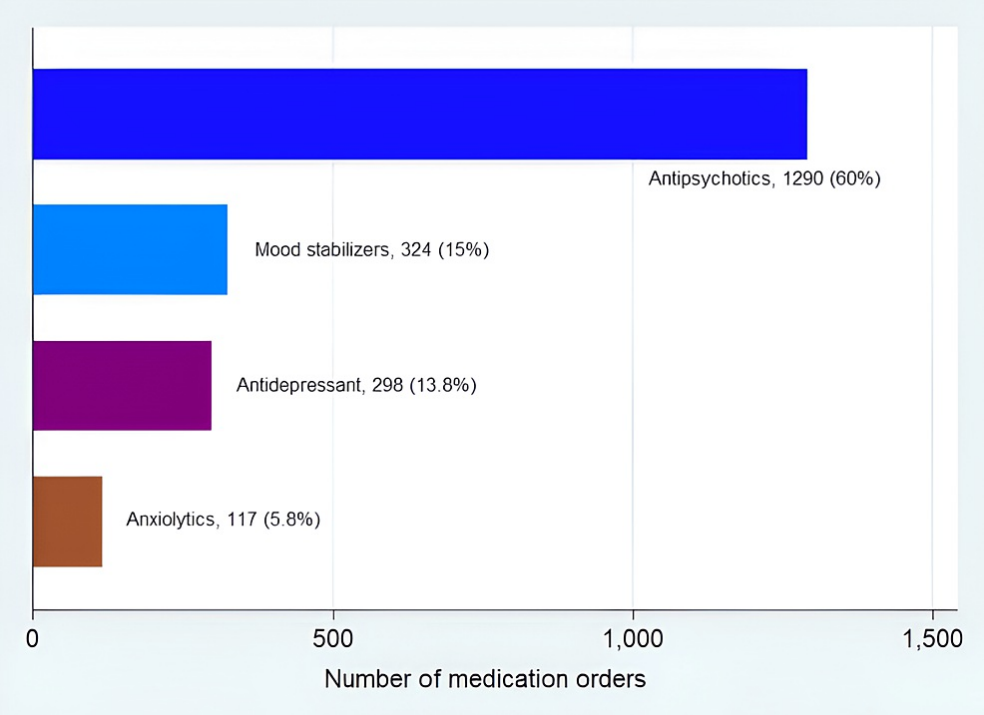
Frequency of medication orders for psychotropic classes

A total of 2,153 medication orders for psychotropic medications were quantified from the medication records (Table 2). SGAs and FGAs were the most commonly prescribed medication classes (1,290, 59.9%), followed by mood stabilizers (324, 15%) (Figure 1). Table 3 shows the pharmacotherapy of psychiatric disorders according to the reported diagnosis. SGAs were the most commonly prescribed medications for the treatment of schizophrenia (15.8%), followed by FGAs (6.7%) and mood stabilizers (3.9%). SGAs were also the top prescribed medication classes for bipolar disorders (8.8%), followed by mood stabilizers (4.4%). 

**Table 2:** Most commonly prescribed psychotropic medications Statistically significant at p-value < 0.05.

Medication		Frequency (%)				Median dose (min-max) mg/day	
		Total n =2153	Outpatient n=837	Inpatient n= 1316	p-value		
Second-generation antipsychotics (SGA)							
Olanzapine		266 (12.4)	99 (4.6)	167 (7.8)	0.001*		10 (5-20)
Quetiapine		248 (11.5)	94 (4.4)	154 (7.2)	0.005*		200 (100-600)
Risperidone		182 (8.5)	77 (3.6)	105 (4.9)	0.504		4 (2-8)
Aripiprazole		59 (2.7)	18 (0.8)	41 (1.9)	0.024*		15 (5-60)
Paliperidone		55 (2.6)	32 (1.5)	23 (1.1)	0.028*		100 (100-150)
Amisulpride		39 (1.8)	15 (0.7)	24 (1.1)	0.446		400 (200-800)
Clozapine		13 (0.6)	3 (0.1)	10 (0.5)	0.119		75 (25-200)
SGA total		862 (40.0)	338 (15.7)	524 (24.3)	0.001*		
First-generation antipsychotics (FGA)							
Haloperidol		168 (7.8)	61 (2.8)	107 (5.0)	0.012*		10 (5-20)
Promethazine HCl		144 (6.7)	56 (2.6)	88 (4.1)	0.125		25 (20-50)
Chlorpromazine HCl		61 (2.8)	26 (1.2)	35 (1.6)	0.780		100 (25-100)
Zuclopenthixol		30 (1.4)	8 (0.4)	22 (1.0)	0.045		200 (200-400)
Flupentixol decanoate		19 (0.9)	16 (0.7)	3 (0.1)	0.001*		20 (20-40)
Sulpiride		6 (0.3)	5 (0.2)	1 (0.0)	0.093		50 (50-100)
FGA total		428 (19.9)	172 (8.0)	256 (11.9)	0.071		
Antidepressant							
Mirtazapine		108 (5.0)	39 (1.8)	69 (3.2)	0.055		30 (15-60)
Escitalopram		73 (3.4)	38 (1.8)	35 (1.6)	0.150		10 (10-20)
Fluoxetine		32 (1.5)	10 (0.5)	22 (1.0)	0.125		20 (20-40)
Citalopram		30 (1.4)	19 (0.9)	11 (0.5)	0.031*		20 (10-20)
Venlafaxine		24 (1.1)	13 (0.6)	11 (0.5)	0.319		75 (75)
Amitriptyline		23 (1.1)	17 (0.8)	6 (0.3)	0.005*		25 (10-50)
Duloxetine		8 (0.4)	1 (0.0)	7 (0.3)	0.083		60 (30-60)
Total antidepressant		298 (13.8)	137 (6.4)	161 (7.5)	0.577		
Mood stabilizers							
Sodium valproate		263 (12.2)	83 (3.9)	180 (8.4)	0.001*		1000 (400-1500)
Carbamazepine		26 (1.2)	15 (0.7)	11 (0.5)	0.158		200 (200-400)
Lamotrigine		18 (0.8)	8 (0.4)	10 (0.5)	0.990		50 (50-200)
Lithium carbonate		17 (0.8)	3 (0.1)	14 (0.7)	0.026		600 (300-600)
Total mood stabilizers		324 (15.0)	109 (5.1)	215 (10.0)	0.001		
Anxiolytics							
Clonazepam		66 (3.1)	19 (0.9)	47 (2.2)	0.007*		2 (1-4)
Midazolam		27 (1.3)	7 (0.3)	20 (0.9)	0.048		10 (7.5-15)
Lorazepam		24 (1.1)	7 (0.3)	17 (0.8)	0.127		2 (1-3)
Total anxiolytics		117 (5.4)	33 (1.5)	84 (3.9)	0.001*		
Anticholinergics							
Benztropine mesylate		124 (5.8)	48 (2.2)	76 (3.5)	0.152		2 (1-2)

**Table 3:** Prescribing patterns of psychotropic medications by common psychiatric disorders FGAs: first-generation antipsychotics, SGAs, second-generation antipsychotics

Medication class	Frequency (%) (n=2153)			
	Total	Outpatient	Inpatient	P value
Schizophrenia				
SGAs	321 (14.9)	135 (6.3)	186 (8.6)	0.451
FGAs	145 (6.7)	72 (3.3)	73 (3.4)	0.201
Mood stabilizers	84 (3.9)	34 (1.6)	50 (2.3)	0.124
Anticholinergics	71 (3.3)	30 (1.4)	41 (1.9)	0.875
Antidepressant	46 (2.1)	22 (1.0)	24 (1.1)	0.897
Anxiolytics	29 (1.3)	8 (0.4)	21 (1.0)	0.045*
Total psychotropic medications	696 (32.3)	301 (14.0)	395 (18.3)	0.380
Bipolar affective disorder				
SGAs	190 (8.8)	61 (2.8)	129 (6.0)	0.568
Mood stabilizers	94 (4.4)	25 (1.2)	69 (3.2)	0.078
FGAs	80 (3.7)	26 (1.2)	54 (2.5)	0.853
Anxiolytics	43 (2.0)	11 (0.5)	32 (1.5)	0.239
Anticholinergic	28 (1.3)	11 (0.5)	17 (0.8)	0.447
Antidepressant	16 (0.7)	8 (0.4)	8 (0.4)	0.078
Total psychotropic medications	451 (20.9)	142 (6.6)	309 (14.4)	0.689
Depression				
Antidepressant	63 (2.9)	45 (2.1)	18 (0.8)	0.740
SGAs	45 (2.1)	26 (1.2)	19 (0.9)	0.015*
FGAs	14 (0.7)	7 (0.3)	7 (0.3)	0.052
Mood stabilizers	9 (0.4)	4 (0.2)	5 (0.2)	0.178
Anxiolytics	7 (0.3)	4 (0.2)	3 (0.1)	0.173
Total psychotropic medications	138 (6.4)	86 (4.0)	52 (2.4)	0.548
Mental retardation				
SGAs	56 (2.6)	38 (1.8)	18 (0.8)	0.527
Mood stabilizers	35 (1.6)	22 (1.0)	13 (0.6)	0.271
FGAs	29 (1.3)	21 (1.0)	8 (0.4)	0.677
Anxiolytics	6 (0.3)	2 (0.1)	4 (02)	0.055
Antidepressant	4 (0.2)	4 (0.2)	0 (0.0)	0.100
Anticholinergic	4 (0.2)	2 (0.1)	2 (0.1)	0.570
Total psychotropic medications	134 (6.2)	89 (4.1)	45 (2.1)	0.312
Anxiety disorders				
Antidepressant	37 (1.7)	24 (1.1)	13 (0.6)	0.186
Anxiolytics	16 (0.7)	10 (0.5)	6 (0.3)	0.628
SGAs	14 (0.7)	8 (0.4)	6 (0.3)	0.837
FGAs	5 (0.2)	4 (0.2)	1 (0.0)	0.100
Mood stabilizers	4 (0.2)	2 (0.1)	2 (0.1)	0.563
Total psychotropic medications	76 (3.5)	48 (2.2)	28 (1.3)	0.235

**Figure 2:**
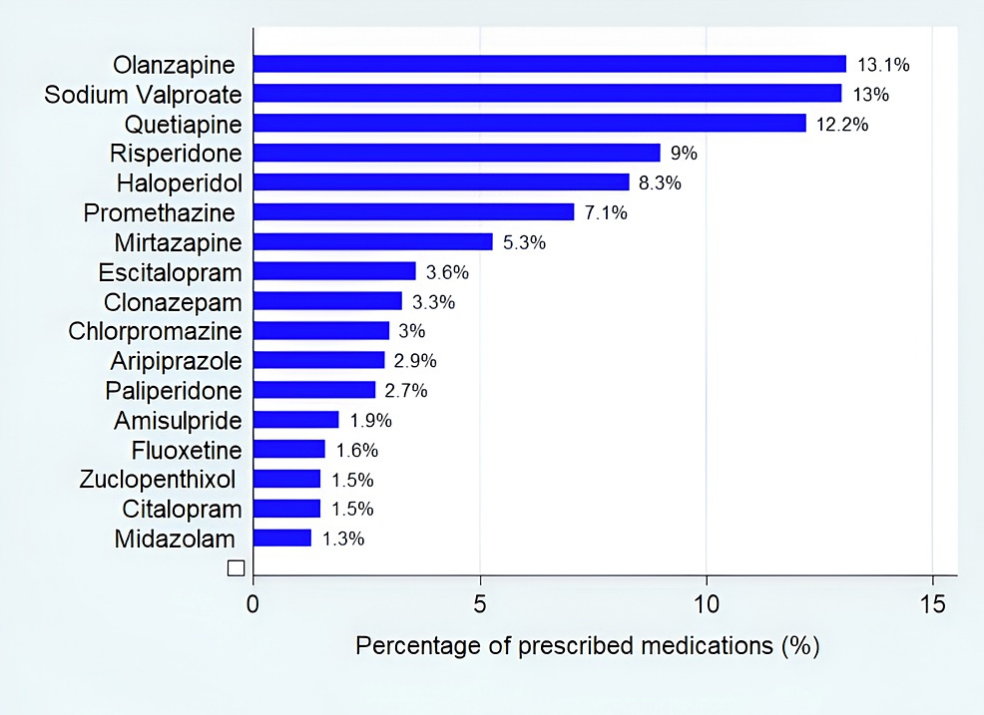
Percentages of the top prescribed psychotropic medications

Olanzapine (266, 12.4%) was the top prescribed SGA, followed by quetiapine (248, 11.5%) and risperidone (182, 8.5%). Haloperidol (168, 7.8%) was the most frequently prescribed FGA, followed by promethazine (144, 6.7%), chlorpromazine (61, 2.8%), and zuclopenthixol (30, 1.4%) (Figure 2).

Sodium valproate was the top prescribed mood stabilizers (263, 12.2%) for the management of bipolar disorders and schizophrenia.

For patients diagnosed with depression, antidepressants were the top prescribed class (2.9%), followed by SGAs (2.1%) (Table 3). The atypical antidepressant mirtazapine (108, 5.0%) was the top prescribed antidepressant, followed by escitalopram (73, 3.4%) from SRRIs.

Anxiolytic medications were commonly prescribed for bipolar disorder (2%), schizophrenia (1.3%), and anxiety disorders (0.7%). Clonazepam (66, 3.1%) was the top prescribed anxiolytic medication. The anticholinergic medication benztropine mesylate was added to the therapeutic regimen for many patients with schizophrenia (3.3%) and bipolar disorder (1.3%). 

Compared to outpatients, SGAs, mood stabilizers, and anxiolytics were significantly more utilized for inpatients (p = 0.001, Table 2) with inpatients more likely to receive multiple medications with a median of four versus three medications for outpatients.

**Figure 3:**
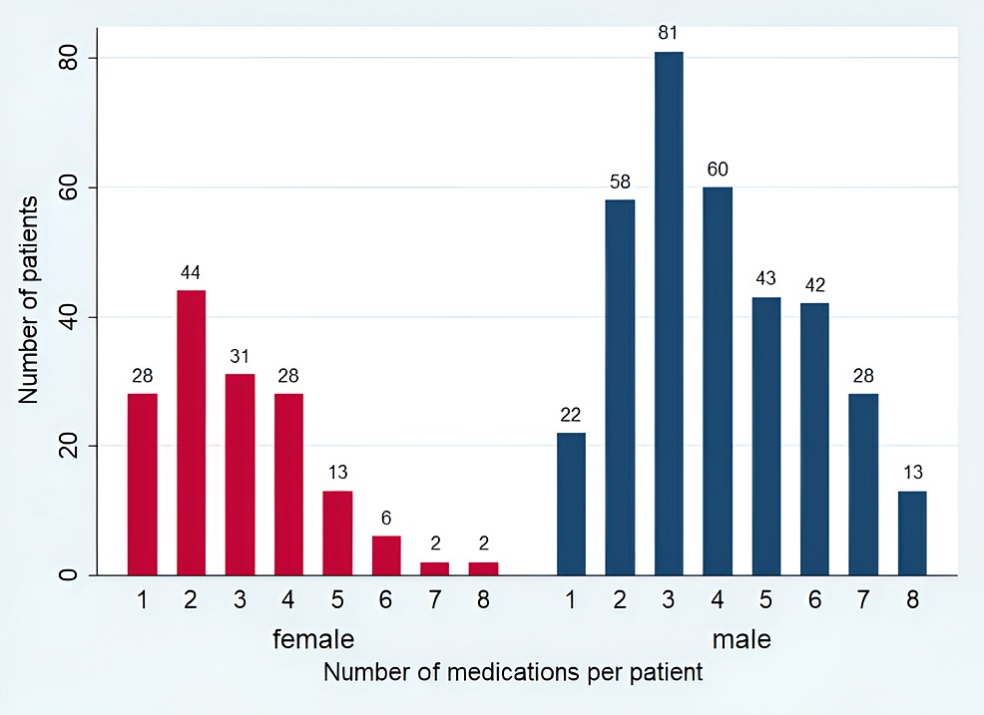
Polypharmacy of psychotropic medications per patient

The majority of patients (471, 89.5%) with different psychiatric diagnoses received more than one psychotropic medication. The median was three prescribed medications per patient and Q1-Q3 ranged from two to five medications (Figure 3). More than one-half of the patients (282, 53.6%) received a combination of antipsychotic and mood stabilizers, 155 (29.5%) received a combination of antipsychotic and antidepressant medications, and 94 (17.9%) of patients received a combination of antipsychotic and anxiolytic medications (Table 4).

**Table 4:** Combination of psychotropic medications

Number of psychotropic medications per patient	Median (Q1, Q3 range)			
	Total	Male	Female	p-value
Antipsychotic	2 (1-3)	3 (2-4)	2 (1-3)	0.001
Antidepressant	1 (0-1)	1 (0-1)	1 (0-1)	0.079
Mood stabilizers	1 (0-1)	1 (0-1)	1 (0-1)	0.665
Antipsychotic + antidepressant	3 (2-4)	3 (2-4)	2 (1-3)	0.004
Antipsychotic + mood stabilizers	3 (2-5)	3 (2-5)	2 (1-3)	0.001
Antipsychotic + anxiolytics	3 (1-4)	3 (2-4)	2 (1-3)	0.003
All psychotropic medications	3 (2-5)	4 (3-5)	3 (2-4)	0.004

## Discussion

This study explored the patterns of psychiatric diseases and the current healthcare practice of prescribing psychotropic medications in a tertiary care mental health hospital in the Tabuk region. However, this study did not investigate the prevalence of mental and behavioral disorders among adult populations as it enrolled only patients who sought mental healthcare services.

The findings revealed that schizophrenia (33.3%) was the most common diagnosis of psychiatric disorders among the study population, followed by bipolar affective disorder. Schizophrenia is a chronic disabling psychiatric disorder with a worldwide age-standardized prevalence of 0.28% in 2016 [16]. The WHO estimated that schizophrenia impacts about 24 million people or one in 300 people (0.32%) worldwide. A higher rate of one in 222 people (0.45%) was screened among adults [17].

The current evidence from the literature revealed sacred research about the prevalence of schizophrenia in Saudi Arabia, except for a few studies that were conducted on specific populations and age groups [18-20]. The Health Statistics Report of the Ministry of Health (MOH) on health activities during the year 2021 summarized a total of 8,434 new and readmitted inpatients and 160,479 new and recurrently attended outpatients with diagnoses of schizophrenia, schizotypal, and delusional disorders [21]. Accordingly, the diagnosis of schizophrenia was the second psychiatric morbidity after mood (affective) disorders, which achieved a total of 190,748 outpatients and 4,788 inpatient cases in the same year. However, the technical report of the large Saudi National Mental Health Survey 2016 focused on anxiety disorders, mood disorders, and disruptive behavior disorders, and it did not report schizophrenia among the prevalent mental health disorders [22]. A recent retrospective study reported on psychotropic medication use among 1,300 elderly outpatients in Jazan quantified 22% of the study population diagnosed with schizophrenia [19].

The second most common psychiatric disorder in this study was bipolar affective disorder (17%). The technical report of the large Saudi National Mental Health Survey 2016 concluded that major depressive disorder was the third most prevalent (6%) mental health condition in Saudi Arabia across a lifetime [4].

This study included 70% of adult males who suffered from psychiatric disorders. Hence, we cannot conclude that there was a higher rate of psychiatric disorders among males compared to females. Similarly, a higher percentage of males were enrolled in previous psychiatric studies in Saudi Arabia [18,23]. This may be attributed to the sociocultural barriers and social stigmatization associated with mental health disorders to seeking healthcare services, especially among females [24]. Consequently, women's mental health is not adequately diagnosed, or their disease is underestimated. Some studies suggested an equal or higher prevalence of psychiatric disorders among females in Saudi Arabia [25]. A study on primary healthcare clinics in Riyadh revealed that 49% of patients, mostly women and highly educated patients, exhibited depressive symptoms [26]. The prevalence of intentional suicide attempts in Saudi Arabia showed that 80% of individuals affected were women from younger age groups due to the absence of a caregiver and low income [27].

This study showed that the age group from 31 to 40 years old was the most common age category prescribed psychotropic medications. Almost similar findings were reported in the Saudi National Mental Health Survey 2016, where mental health disorders were more prevalent in Saudi youth of the age group 15-34 years (40%) [4]. Young age is a stage of life characterized by increasing power, productivity, and adaptation. On the other hand, young adults face the challenges of multidimensional factors, such as unstable employment, frustrating emotional relationships, stressful workloads, and financial worries. Therefore, young adults may develop a loss of mental and behavioral control, especially anxiety and depression, if they experience a lack of optimal physical, cognitive, emotional, and economic resources [28]. 

As per the disease diagnosis, the top prescribed classes of medications were the SGAs (40.0%) and FGAs (20%), primarily for treating schizophrenia and bipolar disorder. Other medications were prescribed in smaller proportions, such as antidepressants (13.8%) and anxiolytics (5.4%). SGAs are more effective for negative symptoms of schizophrenia and cause few or no acutely occurring extrapyramidal adverse effects of FGAs, but they have higher risks for metabolic adverse effects, including weight gain, hyperlipidemia, and diabetes mellitus [1]. However, the evidence of superior efficacy over FGAs is questionable [29]. Even with the optimum utilization of SGAs, achieving symptomatic remission is frequently elusive. Many patients experiencing refractory psychosis will not reach a satisfactory clinical improvement.

Olanzapine followed by quetiapine and risperidone were the top prescribed SGAs. Although clozapine consistently demonstrated clinical efficacy in treatment-resistant schizophrenia, it is not familiar in clinical practice because of adverse effects such as neutropenia [30]. Olanzapine is the safer alternative with similar efficacy to clozapine and the best option for treatment-resistant refractory schizophrenia [31]. The most prescribed initial antipsychotic drugs for adult patients newly diagnosed with schizophrenia in the Jazan region in the south of Saudi Arabia were olanzapine (48.8%), followed by haloperidol (13.9%), aripiprazole (11.3%), and risperidone (10.6%) [18]. Older adults with schizophrenia were frequently prescribed risperidone (11%), olanzapine (7.6%), quetiapine (7.3%), and sulpiride (4.7%) in Jazan [19]. Previous studies conducted on Asian patients with schizophrenia reported that olanzapine was the second most commonly prescribed SGA after risperidone [1]. 

The American Psychiatric Association Practice Guideline (2020) recommends that patients with schizophrenia whose symptoms have improved with an antipsychotic medication should maintain their treatment with the same antipsychotic medication [8].

Haloperidol and promethazine were the top prescribed FGA medications. FGAs cause extrapyramidal effects, and symptomatic recovery is often not maintained despite the appropriate use of these medications [32].

The second prescribed class of medications was mood stabilizers as a combination drug therapy for bipolar affective disorders and schizophrenia. The goal of therapy is to initiate mood-stabilizing medication or optimize the regimen of the current mood stabilizer and then adding a benzodiazepine for short-term adjunctive treatment of agitation or insomnia should be considered if needed. Mood stabilizers were often used as adjunctive therapy to enhance the response to antipsychotic medications for persistent psychotic symptoms, such as aggressive behaviors and disorganized speech and behavior [33,34]. Valproic acid (VPA) is an efficacious medication therapy approved by the U.S. Food and Drug Administration (FDA) for the treatment of acute mania in bipolar disorder usually in combination with antidepressants and antipsychotics [35].

Likewise, the adjunctive use of anticholinergic benztropine mesylate was observed among many patients with schizophrenia and bipolar disorder. This is considered one of the therapy approaches for those experiencing acute dystonia, medication-induced parkinsonism symptoms (e.g., rigidity, tremors, and akinesia), or prophylactic use for patients at a high risk of developing parkinsonism [8,36].

Our findings showed that mirtazapine and SSRIs were most commonly prescribed for treating depression. Mirtazapine was a preferred choice for the study population because it was effective in improving both signs of depression and insomnia in clinical practice. SGAs, such as SSRIs, are usually recommended as initial therapy for depression because of superior efficacy and better tolerability [37]. Nevertheless, miscellaneous classes of antidepressants, such as mirtazapine, have been increasingly prescribed recently, taking into account clinical patient considerations, efficacy, and tolerability [38].

In this study, prescribing benzodiazepines, such as lorazepam and clonazepam, was consistent with the 2020 American Psychiatric Association Practice Guideline recommendation as one of the treatment options for patients who experience akathisia associated with antipsychotic therapy. Another option for treating akathisia is the β-adrenergic blocking agent propranolol [8].

Regarding polypharmacy, our findings revealed that more than one-half of patients (53.6%) received a combination of antipsychotics and mood stabilizers, particularly patients who were diagnosed with schizophrenia and bipolar disorder. Three medications were the median number of prescribed psychotropic drugs per patient. The treatment plan for psychiatric disorders depends on concurrent symptoms (e.g., depression, hopelessness, hostility, and impulsivity) and concurrent diagnoses, such as depression, alcohol use disorder, and other substance use disorders. Polypharmacy was commonly observed in patients diagnosed with schizophrenia and bipolar disorders because of prescribing adjunctive therapy, such as mood stabilizers and anticholinergics in combination with the mainstay medications.

In summary, guidelines sometimes do not entirely reflect routine clinical practices. This study demonstrated that the prescribing of psychotropic medications in the mental health hospital was markedly aligned with recent evidence-based recommendations of the current literature. Physicians should be cautious about the appropriate indications, effectiveness, and potential side effects when prescribing psychotropic medications. Adhering to the prescribing guidelines is highly recommended in clinical practice to optimize the therapeutic management of psychiatric diseases and eliminate avoidable harm to patients. A multidisciplinary healthcare team, involving a psychiatrist, a pharmacist, and a nurse, should collaborate closely in the care of patients. The input of pharmacist interventions is strongly recommended to reduce polypharmacy and ensure patient safety.

We hope the findings will contribute to the current literature and expand the knowledge about the trends in antipsychotic prescribing in one of the main regions of Saudi Arabia in an attempt to continue building a national database to guide future steps toward improving the health system and overall patient outcomes.

Study limitations

Because of the retrospective nature of the study design, the analysis encountered challenges related to the missing data in recording adverse drug reactions and results of laboratory investigations, especially for outpatients. Missing data on recording patient-related factors can affect the use of psychotropic medication. The study setting is specifically for the management of mental health diseases, and thus, the use of non-psychotropic drugs was poorly documented. There was a lack of data documenting pharmacist interventions for modifying drug-related problems during the prescribing process.

## Conclusions

This study revealed that schizophrenia and bipolar disorders were common mental health disorders in adults who sought mental healthcare. The psychotropic prescription patterns for adult patients were broadly consistent with the practice guidelines. The mainstay and adjunctive medication therapy for psychiatric disorders depended on concurrent symptoms. Pharmacists-led interventions, including medication reviews and regular monitoring of psychotropic prescriptions, are crucial to monitor inappropriate psychotropic polypharmacy.

## Appendices

https://forms.gle/yQduqTnSaBGAsuN29
